# Diabetes Mellitus, Nonalcoholic Fatty Liver Disease, and Conjugated Linoleic Acid (Omega 6): What Is the Link?

**DOI:** 10.1155/2019/5267025

**Published:** 2019-04-08

**Authors:** Mona Hegazy, Naglaa M. Elsayed, Hala M. Ali, Hanan G. Hassan, Laila Rashed

**Affiliations:** ^1^Internal Medicine Department, Faculty of Medicine, Cairo University, Egypt; ^2^Biochemistry Department, Faculty of Medicine, Cairo University, Egypt

## Abstract

**Background and Objective:**

Type 2 DM and obesity are the coming epidemics and their association with NAFLD is well established; essential fatty acids are vital for body health yet the body cannot make them; 2 essential fatty acids are especially important: linoleic (omega-6) and alpha-linoleic (omega-3) acids; they can be considered as “bioactive lipids” and serve as functional foods.

**Methods:**

50 type 2 Egyptian diabetic patients controlled on oral hypoglycemic drugs together with 20 age- and sex-matched healthy participants were enrolled in the study; all were subjected to complete history taking, BMI, fasting plasma glucose, HOMA-IR, ALT, AST, GGT, urea and creatinine, total lipid profile, hepatitis markers including hepatitis B surface antigen and hepatitis C virus antibodies, conjugated linoleic fatty acid “CLA,” and abdominal ultrasound for grading of NAFLD.

**Results:**

Our study in Egyptian diabetics with NAFLD revealed a low level of serum CLA compared to healthy control; such deficiency was more marked with advanced grades of NAFLD; lowest levels were observed in those with severe steatosis (NASH) with definite association between CLA and obesity.

**Conclusion:**

Insulin resistance is the main link between NAFLD, diabetes, and obesity. Conjugated linoleic acid (CLA) has a role in fat deposition in the liver and in development and improvement of insulin resistance. Fatty food had a documented role in the pathogenesis of obesity and diabetes but it can also be the cure.

## 1. Introduction

Some important food components cannot be made by the body and have to be supplied from outside, and they are vital for human health and are so-called “essential”; of those 2, main polyunsaturated fatty acids are getting much attention: linoleic acid (omega 6) and alpha-linoleic acid (omega 3) can modulate metabolism and alter body weight so they can be considered as bioactive lipids and can serve as functional food [1].

Conjugated linoleic acid (CLA) refers to a heterogeneous group of geometric and constitutional isomers of linoleic acid. Up to 28 forms are known differing from each other in their double-bond arrangement; of them, “c9,t11” and “t10,c12” are especially important [1].

CLA is a natural *trans* fat present in many healthy foods, e.g., animal food sources mainly ruminant animals such as cows, sheep, and goats with greatly variable amounts depending on the animals' feeding pattern; e.g., grass-fed cow dairy and beef have 300-500% higher CLA content (mainly c9,t12) compared to grain-fed cows [2], while industrial *trans* fats found mainly in chemically treated vegetable oils, e.g., sunflower oils, are harmful and supplements are mainly rich in t10,c12 isomer of CLA that does not have the same health effects as this taken from natural foods [3].

The liver is the main organ concerned with energy homeostasis converting excess glucose into fatty acids (FA) which later export as triglycerides (TG) [4]. It is also an important target for CLA effects regardless of its physiological condition.

Nonalcoholic fatty liver disease (NAFLD) is an important health concern mainly due to its high global prevalence (20-30% of adult population) especially in subjects having insulin resistance and metabolic syndrome [5]. Hepatic lipids in the form of triglycerides primarily accumulate in the liver [6] with multiple potential steps involved in the progression of NAFLD including increased circulating fatty acid uptake [7], enhanced de novo lipogenesis [8], diminished rate of FA oxidation [9], or reduced secretion [10].

NAFLD is a major cause of chronic liver disease and it encompasses a spectrum from simple steatosis to steatohepatitis, fibrosis, or cirrhosis. The mechanisms involved in the occurrence of NAFLD and its progression are probably due to the metabolic profile expressed within the context of a genetic predisposition and is associated with higher energy intake [11].

The metabolic syndrome is a cluster of alterations associated with an increased risk for the development of cardiovascular diseases and diabetes. NAFLD patients have more than a feature of the metabolic syndrome, and now they are considered the hepatic component of metabolic syndrome. Several scientific advances in understanding the association between NAFLD and MS have identified insulin resistance as the key aspect in the pathophysiology of both diseases. In the multiparallel hit theory of NAFLD pathogenesis, IR was described to be central in the predisposition of hepatocytes to be susceptible to other multiple pathogenetic factors [12].

Despite the absence of specific guidelines for treatment of NAFLD, most recommendations advise body weight reduction [13]; the dietary fatty acid composition is crucial in NAFLD development as 15% of hepatic TG comes from the diet [14]; in this regard, bioactive lipid is important.

### 1.1. Objective

The aim of the present study is to find out a relation between obesity, insulin resistance, and conjugated linoleic fatty acid in type 2 diabetic patients with variable degrees of NAFLD severity.

## 2. Patients and Methods

A total of 50 type 2 diabetic patients, 25 normal weight, and 25 obese (36 females and 14 males) with NAFLD were enrolled in this prospective study from December 2015 to November 2016. They were recruited from Diabetes and Hepatology outpatient clinics of Kasr El Ainy Hospital. Their age ranged from 29 to 57 years.

Twenty healthy, normal-weight volunteers who were age- and sex-matched were included as a control group. An informed consent was obtained from all participants prior to enrolment.

The diagnosis of NAFLD was based on ultrasonographic finding of a bright liver, which was defined and graded as a diffuse hyperechoic echotexture (bright liver) (grade 1), increased liver echotexture compared to the kidney (grade 2), vascular blurring (grade 3), and deep attenuation (grade 4) [15].

The study was conducted with appropriate approval by the Ethics Committee of Cairo University (N-17-2015) in accordance with the ethical guidelines of the Declaration of Helsinki [16].

### 2.1. Inclusion Criteria

For diabetic patients with NAFLD, all were controlled on oral hypoglycemic medications, age above 18 years, normal and overweight (BMI > 25 kg/m^2^), and a bright liver on abdominal ultrasound with or without elevated liver enzymes.

### 2.2. Exclusion Criteria

Patients were excluded from the study if one of the following criteria was present: any liver disease other than NAFLD such as hepatitis B or C, autoimmune hepatitis, alpha-one antitrypsin deficiency or Wilson's disease, alcohol consumption, history of drug intake (such as use of amiodarone, corticosteroids, tamoxifen, methotrexate, and oral contraceptives), pregnancy, hypertension, thyroid disease, malignancy, history of taking any omega-6 or CLA capsules, and decompensated liver disease [17]. Any subjects with evidence of local or systemic infection on physical examination were also excluded from the study.

### 2.3. Selection of Control

In all healthy control volunteers, the absence of any current or past liver disease was established based on the presence of normal liver function tests and the presence of a normal abdominal ultrasound.

### 2.4. Methodology

All participants included in the study were subjected to the following: detailed history taking, complete clinical examination including anthropometric evaluation (weight, height, and body mass index (BMI) were calculated). BMI between 25 and 29.9 kg/m^2^ and ≥30 kg/m^2^ were defined as overweight and obesity, respectively.

Laboratory investigations include fasting plasma glucose “oxidase method,” ELISA for conjugated linoleic fatty acid “CLA,” fasting insulin level, calculation of HOMA to determine insulin resistance (American formula: fasting glucose ^“^mg/dl^”^ × fasting insulin ^“^*μ*U/ml^”^/405) [18], liver function tests, serum transaminases (ALT, AST), GGT, urea and creatinine, total lipid profile, and hepatitis markers including hepatitis B surface antigen and hepatitis C virus antibodies.

Abdominal ultrasonography was performed for all subjects using a Toshiba Aplio XV scanner equipped with a broadband 3.5 MHz curved array probe to assess the presence of liver steatosis (bright liver) and by a single operator to avoid interobserver variability. Patients were examined after at least 8 hours of fasting and were examined in the supine, right, and left lateral positions.

### 2.5. Statistical Methods

Data were statistically analyzed using the General Linear Model (GLM) procedure of SAS software (SAS Institute, version 8.2, 2001) and Excel 2010. Differences between groups were evaluated by one way ANOVA. The significant differences between group means were tested by the Duncan multiple range test. The data were presented in mean ± SE. Level of significance was set at <0.05.

## 3. Results

The anthropometric and laboratory data of all enrolled patients and control are shown in Table 1; diabetics have statistically significant lower CLA levels (13.25 ± .0.66) compared to control (9.78^b^ ± 1.34), with markedly low levels in obese diabetics (6.13^c^ ± 0.57).

According to results of abdominal ultrasound examination, diabetic patients were classified into 4 groups according to the grade of NAFLD: grade 1 steatosis (n.4), grade II steatosis (n.16), grade III steatosis (n.18), and grade IV steatosis (n.12).

**Figure 1:**
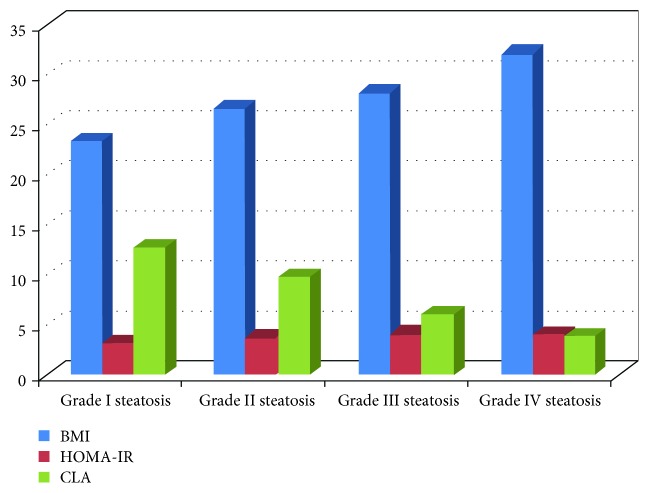
Comparison between BMI, HOMA-IR, and CLA of all enrolled subjects according to their grade of steatosis. BMI: body mass index; CLA: conjugated linoleic acid.

All diabetic subjects with different grades of NAFLD were compared as regards their BMI and different laboratory values, and comparative data are illustrated in Table 2, and data are presented graphically in Figure 1.

All patients with grade I steatosis had normal BMI, and all grade IV patients were obese (high BMI) as they represent the extremes; therefore, no comparison—as regards laboratory data including their level of CLA—was done among these two grades. While in those with grades II and III NAFLD, some of the patients had normal BMI while the others were obese so a comparison was done in those patients with grades II and III steatosis. Data of patients with grade II NAFLD after being classified according to their BMI is illustrated in Table 3.

Diabetic patients with grade III NAFLD also have been classified into 2 groups according to their BMI: diabetics with normal BMI (8 patients) and diabetics with high BMI (10 patients), and comparison was done and is illustrated in Table 4.

## 4. Discussion

Type 2 diabetes is a major health concern with an expected doubling of the number of patients in the next few years; insulin resistance plays an important role in the pathogenesis of type 2 diabetes [19].

NAFLD nowadays is ranked as the commonest chronic liver disease as its prevalence is rising: estimated 20-30% in the general population, and can reach up to 70-90% in obese and diabetic patients [20]. It has a strong association with many conditions; most commonly obesity, type 2 diabetes, insulin resistance, dyslipidemia, and hypertension, and it is considered to be “hepatic manifestation of metabolic syndrome” [21].

And despite that no specific guidelines exist concerning the treatment of NAFLD, yet most recommendations were aimed at treating associated conditions such as obesity by weight reduction and diabetes by glycemic control and lipid control [22]. In this regard, modulating body weight and metabolism through functional foods as bioactive lipids may be important. A specific group of polyunsaturated FA collectively known as conjugated linoleic acid (CLA) has been suggested to have an effect on regulating energy metabolism.

This polyunsaturated conjugated fatty acid is a naturally present *trans* fat found mainly in dairy products (milk, cheese, yogurt, and cream) and meat, veal, and lamb from ruminant grass-fed animals. It is formed as an intermediate during isomerization of dietary linoleic acid by the action of bacteria in the digestive tract of ruminant animals [23], and it is formed mainly of *cis*-9,*trans*-11 CLA (>80%) with small amounts of (t10,c12) CLA and other isomers.

It is also possible to obtain CLA in an industrial (commercial) form, through the partial hydrogenation of linoleic acid or by thermal treatments, aiming at producing a compound with a maximum biological activity and with a defined chemical composition; it consists of equal amounts of (c9,t11) CLA and t10,*cis*-12 CLA and other isomers [24].

The main organ concerned with energy homeostasis is the liver, It is also an important target for CLA effects that differ according to the type of isomer; e.g., in animals, *trans*-10,*cis*-12 caused increased lipid accumulation and hepatic steatosis but neither *trans*-10,*cis*-12 nor *cis*-9,*trans*-11 CLA has an effect on atherosclerosis [25]; both isomers promote insulin sensitivity by reducing adipose inflammation [26], and both also protect against oxidative stress and enhance hepatic mitochondrial function decreasing insulin resistance in rats by both isomers [27].

The relation between type 2 diabetes and NAFLD is well established; there is an increased risk of NAFLD among patients with type 2 diabetes and despite as previously mentioned that the pathogenesis of NAFLD is not fully understood, yet the most accepted theory is “multiple hit theory,” and the first hit being insulin resistance as the most important contributing factor with obesity as the main insulin-resistant state causing inflammatory state and altering lipid metabolism [28, 29].

On the other hand, NAFLD also can act as a risk factor for development of diabetes; a study evaluated subjects with NAFLD and control subjects for 11 years; none of them has diabetes at baseline; those with NAFLD were more likely to develop metabolic syndrome and diabetes [30], and when those patients develop overt diabetes in the setting of preceding obesity and insulin resistance, it was found that diabetes acts as an independent risk factor for the progression of NAFLD [31].

So obesity and insulin resistance are both initiating and stimulating risk factors for the development and progression of NAFLD, hence the importance of dietary modifications and functional foods as a tool in prevention and management.

Conjugated linoleic acid (CLA) is a natural polyunsaturated fatty acid. It is the most common omega 6 fatty acid widely used for slimming; conjugated refers to the arrangement of double bonds in the fatty acid molecule (mainly *cis* and *trans*), and the number refers to the placement of such bonds in the chain [32]; there are nearly 28 different isomers.

CLA is a natural *trans* fat present in many healthy foods, e.g., animal food sources mainly ruminant animals such as cows, sheep, and goats with greatly variable amounts depending on the animals' feeding pattern; e.g., grass-fed cow dairy and beef have 300-500% higher CLA content (mainly c9,t12) compared to grain-fed cows, while industrial *trans* fats found mainly in chemically treated vegetable oils, e.g., sunflower oils, are harmful and supplements are mainly rich in t10,c12 isomer of CLA that does not have the same health effects as this taken from natural foods.

Researchers have discovered multiple biological activities of such polyunsaturated fatty acid in animals and in humans including antiobesity [33], reduction of body fat mass [34], improving body composition, and increasing muscle mass [35] explaining its wide use in slimming and body configuration.

Also, some studies show that in areas where people consume large amounts of CLA from natural sources, the risk of developing diseases like type 2 diabetes and cancer is low [36], and those from countries where cows are grass-fed have low risk of heart disease claimed to high CLA content and other substances in their foods [37]. CLA was found to exert anti-inflammatory effects [1].

To the best of our knowledge, this is the first study to measure CLA levels in human and it revealed that CLA (c9,t12 is the form mainly assessed as none of the patients has taken supplementation throughout their life) was significantly lower in diabetic obese patients (6.1 ± 0.57 ng/ml) compared to diabetic nonobese patients (9.78 ± 1.34 ng/ml) and also when compared to healthy control participants (13.25 ± 0.66 ng/ml) with a *P* value of <0.0001.

Regarding NAFLD subgroups, CLA was progressively lower as the grade of steatosis advances: lowest in grade IV (NASH).

These data although on a small group of Egyptian obese diabetics raise a query concerning the role of supplements in those patients with documented deficiency whether to give or not and which type.

Research into the previous studies of CLA supplementation in humans revealed contradictory results about the benefits and possible health hazards of such supplementations if any.

Some studies of supplementation with CLA together with low-dose vitamin E and low-calorie diet in subjects with NAFLD found improved body composition, serum HbA1c, oxidative stresses, and lipid profile [38]. It also showed decreased insulin resistance through increased adiponectin production and increasing PPAR*γ* expression, while other studies, especially in humans, showed no effect on lipid profile or glycemic status [39, 40] despite moderate hyperinsulinemia.

The discrepancy of results may be related to multiple factors among which are study population, length of the intervention period, type of CLA isomers given, and the association with lifestyle modifications including exercise or low-calorie diet.

Some studies involved 4-8 up to 12 weeks of supplementation, i.e., short periods, while others reported not less than 6 months of use and revealed statistically significant yet clinically irrelevant decrease of body weight affecting mainly body fat mass and BMI while no significant effect on waist circumference, and these changes were mainly observed in obese or overweight individuals [41].

Concerning the type of isomer given, *cis*-9,*trans*-11 CLA (which is the type mostly present in natural food) was shown to promote insulin sensitivity by reducing adipose inflammation. It mainly enhances hepatic mitochondrial function and conveys protection against oxidative stress through action on mitochondrial antioxidant enzymes increasing their activities while of the different CLA isomers, *trans*-10,*cis*-12 CLA (which is taken in supplement) causes increased lipid accumulation mainly through reduction of adiponectin and leptin leading to hepatic steatosis [42].

## 5. Conclusion and Limitation and Recommendations

- The study is a cross-sectional study with a small number of patients in the subgroups due to the cost of kits; it revealed a low level of serum CLA compared to healthy control; such deficiency was more marked with advanced grades of NAFLD with a definite association between CLA and obesity
- We recommend with this documented deficiency a study on a large scale of patients to document such deficiency and to try supplementation with CLA to prove causality not only an association; mainly in consumption of foods naturally enriched with CLA, we recommend against artificial supplements due to possible harmful effects
- Listed here are some of the functional diet-rich sources with CLA as we recommend incorporating them in the typical Egyptian diet so as to modulate the level of CLA: namely, grass-fed beef, whole milk cheddar cheese from grass-fed cows, and whole milk from grass-fed cows and lamb.

## Figures and Tables

**Table 1:** BMI and laboratory data of enrolled subjects.

Parameter	Control (n.20)	Diabetics with normal BMI (n.25)	Diabetics with high BMI (n.25)	*P* value
BMI	24.02^b^ ± 0.05	24.21^b^ ± 0.14	30.67^a^ ± 0.83	**<0.0001** ^∗∗^
AST (IU/l)	18.70^b^ ± 1.62	20.56^b^ ± 0.52	31.84^a^ ± 1.32	**<0.0001** ^∗∗^
ALT (IU/l)	19.10^c^ ± 1.45	32.12^b^ ± 1.00	54.32^a^ ± 1.41	**<0.0001** ^∗∗^
T cholesterol (mg/dl)	188.80^b^ ± 4.91	192.52^b^ ± 4.62	215.04^a^ ± 7.52	**<0.0051** ^∗^
HDL (mg/dl)	29.05^a^ ± 0.87	18.64^b^ ± 0.52	16.72 ± 0.41	**<0.0001** ^∗∗^
LDL (mg/dl)	141.00^b^ ± 4.53	144.68^b^ ± 4.82	155.76^a^ ± 5.38	**<0.1026**
TG (mg/dl)	94.10^c^ ± 3.92	173.84^b^ ± 13.69	223.28^a^ ± 15.89	**<0.0001** ^∗∗^
FBG (mg/dl)	110.15^c^ ± 3.65	291.42^b^ ± 20.84	223.72^a^ ± 13.04	**<0.0001** ^∗∗^
Insulin (IU/ml)	6.28^b^ ± 0.14	7.33^a^ ± 0.33	7.37^a^ ± 0.37	**<0.0300** ^∗^
HOMA-IR	1.72^b^ ± 0.08	4.00^a^ ± 0.27	3.79^a^ ± 0.23	**<0.0001** ^∗∗^
CLA (ng/ml)	13.25^a^ ± 0.66	9.78^b^ ± 1.34	6.13^c^ ± 0.57	**<0.0001** ^∗∗^

^a,b,c^Means ± SE in the same row with different superscripts are significantly different. ^∗∗^Highly significant. ^∗^Significant; AST: aspartate transaminase; ALT: alanine aminotransferase; T cholesterol: total cholesterol: HDL; high-density lipoprotein: LDL; low-density lipoprotein; TG: triglycerides; FBG: fasting blood glucose; HOMA-IR: homeostatic model assessment insulin resistance; CLA: conjugated linoleic acid.

**Table 2:** Comparison between BMI and laboratory data of all studied diabetic patients according to their grade of NAFLD.

Parameter	Grade I (n.4)	Grade II (n.16)	Grade III (n.18)	Grade IV (n.12)	*P* value
BMI	23.4^c^ ± 0.88	26.73^b^ ± 0.98	28.18^b^ ± 1.16	32.04^a^ ± 1.25	**<0.0001** ^∗∗^
AST (IU/l)	19.75^d^ ± 1.35	25.75^c^ ± 1.45	29.62^b^ ± 1.00	34.50^a^ ± 1.32	**<0.0001** ^∗∗^
ALT (IU/l)	27.75^d^ ± 2.03	39.00^c^ ± 2.52	46.69^b^ ± 2.32	53.12^a^ ± 2.36	**<0.0001** ^∗∗^
T. Chol (mg/dl)	203.0^d^ ± 4.91	219.63^c^ ± 4.62	237.88^b^ ± 7.52	251.56^a^ ± 7.52	**<0.0324** ^∗^
HDL (mg/dl)	17.50^a^ ± 0.57	16.94^b^ ± 0.62	14.5^c^ ± 0.44	13.19^c^ ± 0.41	**<0.0039** ^∗^
LDL (mg/dl)	156.75^c^ ± 2.56	164.44^b^ ± 3.02	176.56^a^ ± 2.38	179.63^a^ ± 3.63	<0.0210
TG (mg/dl)	145.00^d^ ± 14.92	197.06^c^ ± 15.89	235.75^b^ ± 15.89	295.06^a^ ± 8.89	**<0.0001** ^∗∗^
FBG (mg/dl)	209.25^**b**^ ± 10.66	210.56^**b**^ ± 15.07	223.72^**a**^ ± 13.04	248.94^**a**^ ± 10.04	<0.3011
Insulin (*μ*IU/ml)	6.40^bc^ ± 0.21	7.39^a^ ± 0.29	6.98^ab^ ± 0.24	6.11^c^ ± 0.37	**0.0033** ^∗^
HOMA-IR	3.22^b^ ± 0.16	3.81^a^ ± 0.23	4.21^a^ ± 0.18	4.10^a^ ± 0.34	**<0.0162** ^∗^
CLA (ng/ml)	12.80^a^ ± 0.54	9.92^b^ ± 0.76	6.16^c^ ± 0.62	3.91^d^ ± 0.57	**<0.0001** ^∗∗^

**Table 3:** Comparison between laboratory data of subjects with grade II NAFLD after being classified according to their BMI into those with normal BMI and with high BMI.

Parameter	Diabetics with normal BMI (n.5)	Diabetics with high BMI (n.11)	*P* value
BMI (kg/m^2)^	24.21 ± 0.14	30.8 ± 0.75	<0.0001^∗∗^
AST (IU/l)	21.10 ± 1.36	34.30 ± 1.62	<0.0001^∗∗^
ALT (IU/l)	33.60 ± 2.34	55.6 ± 3.31	<0.0001^∗∗^
T. Chol (mg/dl)	214.10 ± 5.24	225.20 ± 7.42	<0.0056^∗∗^
HDL (mg/dl)	18.1 ± 0.61	16.10 ± 0.43	<0.04500^∗^
LDL (mg/dl)	163.2 ± 3.69	164.2 ± 2.61	<0.2135
TG (mg/dl)	177.6 ± 13.05	221.8 ± 14.45	<0.0001^∗∗^
FBG (mg/dl)	199.8 ± 13.36	228.80 ± 14.89	<0.1174
Insulin (*μ*IU/ml)	7.62 ± 0.34	7.10 ± 0.42	0.2875
HOMA-IR	3.78 ± 0.26	3.92 ± 0.32	<0.4188
CLA (ng/ml)	8.42 ± 0.58	7.92 ± 0.65	<0.3569

**Table 4:** Comparison between laboratory data of studied diabetics with grade III NAFLD.

Parameter	Diabetics with normal BMI (n.8)	Diabetics with high BMI (n.10)	*P* value
BMI (kg/m^2^)	24.21 ± 0.14	30.9 ± 0.83	**<**0.0001^∗∗^
AST (IU/l)	20.80 ± 0.84	33.80 ± 1.18	<0.0001^∗∗^
ALT (IU/l)	34.70 ± 1.91	53.60 ± 2.69	<0.0001^∗∗^
T. Chol (mg/dl)	224.4 ± 5.17	243.3 ± 7.32	<0.0188^∗^
HDL (mg/dl)	15.1 ± 0.45	13.9 ± 0.64	<0.0806
LDL (mg/dl)	107.7 ± 3.39	177.8 ± 5.64	<0.2244
TG (mg/dl)	194.7 ± 12.31	260.3 ± 15.41	<0.0001^∗∗^
FBG (mg/dl)	251.6 ± 14.6	266.7 ± 17.32	<0.4933
Insulin (*μ*IU/ml)	7.34 ± 0.28	6.46 ± 0.42	0.2976
HOMA-IR	4.73 ± 0.28	3.95 ± 0.38	<0.0681
CLA (ng/ml)	7.78 ± .0.26	5.92 ± 0.37	<0.0002^∗∗^

## Data Availability

The data used to support the findings of this study are available from the corresponding author upon request.

## References

[1] Ching C. K. (2007). *Fatty Acids in Foods and Their Health Implications*.

[2] Dhiman T. R., Anand G. R., Satter L. D., Pariza M. W. (1999). Conjugated linoleic acid content of milk from cows fed different diets. *Journal of Dairy Science*.

[3] Bissonauth V., Chouinard Y., Marin J., Leblanc N., Richard D., Jacques H. (2006). The effects of t10,c12 CLA isomer compared with c9,t11 CLA isomer on lipid metabolism and body composition in hamsters. *The Journal of Nutritional Biochemistry*.

[4] Dey A., Chandrasekaran K. (2009). Hyperglycemia-induced changes in liver: in vivo and in vitro studies. *Current Diabetes Reviews*.

[5] Fabbrini E., Sullivan S., Klein S. (2010). Obesity and nonalcoholic fatty liver disease: biochemical, metabolic, and clinical implications. *Hepatology*.

[6] Amarapurkar D. N., Patel N. D. (2004). Clinical spectrum and natural history of non-alcoholic steatohepatitis with normal alanine aminotransferase values. *Tropical Gastroenterology*.

[7] Reddy J. K., Sambasiva Rao M. (2006). Lipid metabolism and liver inflammation. II. Fatty liver disease and fatty acid oxidation. *American Journal of Physiology-Gastrointestinal and Liver Physiology*.

[8] Diraison F., Moulin P., Beylot M. (2003). Contribution of hepatic de novo lipogenesis and reesterification of plasma non esterified fatty acids to plasma triglyceride synthesis during non-alcoholic fatty liver disease. *Diabetes & Metabolism*.

[9] Ronis M. J., Chen Y., Jo C.-H., Simpson P., Badger T. M. (2004). Diets containing soy protein isolate increase hepatic CYP3A expression and inducibility in weanling male rats exposed during early development. *The Journal of Nutrition*.

[10] Huang H.-L., Lin W.-Y., Lee L.-T., Wang H.-H., Lee W.-J., Huang K.-C. (2007). Metabolic syndrome is related to nonalcoholic steatohepatitis in severely obese subjects. *Obesity Surgery*.

[11] Abenavoli L., Milic N., di Renzo L., Preveden T., Medić-Stojanoska M., de Lorenzo A. (2016). Metabolic aspects of adult patients with nonalcoholic fatty liver disease. *World Journal of Gastroenterology*.

[12] (2016). EASL–EASD–EASO Clinical Practice Guidelines for the management of non-alcoholic fatty liver disease. *Journal of Hepatology*.

[13] Zivkovic A. M., German J. B., Sanyal A. J. (2007). Comparative review of diets for the metabolic syndrome: implications for nonalcoholic fatty liver disease. *The American Journal of Clinical Nutrition*.

[14] Ferramosca A., Zara V. (2014). Modulation of hepatic steatosis by dietary fatty acids. *World Journal of Gastroenterology*.

[15] Ricci C., Longo R., Gioulis E. (1997). Noninvasive in vivo quantitative assessment of fat content in human liver. *Journal of Hepatology*.

[16] World Medical Association (2013). World Medical Association Declaration of Helsinki: ethical principles for medical research involving human subjects. *JAMA*.

[17] Hegazy M. A., Samie R. M. A., Ezzat A., Ramadan N., Rashed L. A., ElSayed A. M. (2016). PNPLA3 and TNF-*α* G238A genetic polymorphisms in Egyptian patients with different grades of severity of NAFLD. *Open Journal of Gastroenterology*.

[18] Tarantino G., Colicchio P., Conca P. (2009). Young adult obese subjects with and without insulin resistance: what is the role of chronic inflammation and how to weigh it non-invasively?. *Journal of Inflammation*.

[19] Wild S., Roglic G., Green A., Sicree R., King H. (2004). Global prevalence of diabetes: estimates for the year 2000 and projections for 2030. *Diabetes Care*.

[20] Targher G., Day C. P., Bonora E. (2010). Risk of cardiovascular disease in patients with nonalcoholic fatty liver disease. *The New England Journal of Medicine*.

[21] McCullough A. J. (2011). Epidemiology of the metabolic syndrome in the USA. *Journal of Digestive Diseases*.

[22] de Piano A., Prado W. L., Caranti D. A. (2007). Metabolic and nutritional profile of obese adolescents with nonalcoholic fatty liver disease. *Journal of Pediatric Gastroenterology and Nutrition*.

[23] Kepler C. R., Tucker W. P., Tove S. B. (1970). Biohydrogenation of unsaturated fatty acids. IV. Substrate specificity and inhibition of linoleate delta 12-cis, delta-11-trans-isomerase from Butyrivibrio fibrisolvens. *Journal of Biological Chemistry*.

[24] Chin S. F., Liu W., Storkson J. M., Ha Y. L., Pariza M. W. (1992). Dietary sources of conjugated dienoic isomers of linoleic acid, a newly recognized class of anticarcinogens. *Journal of Food Composition and Analysis*.

[25] Cooper M. H., Miller J. R., Mitchell P. L., Currie D. L., McLeod R. S. (2008). Conjugated linoleic acid isomers have no effect on atherosclerosis and adverse effects on lipoprotein and liver lipid metabolism in apoE^−/−^ mice fed a high-cholesterol diet. *Atherosclerosis*.

[26] Reynolds C. M., Roche H. M. (2010). Conjugated linoleic acid and inflammatory cell signalling. *Prostaglandins, Leukotrienes and Essential Fatty Acids*.

[27] Choi J. S., Koh I.-U., Jung M. H., Song J. (2007). Effects of three different conjugated linoleic acid preparations on insulin signalling, fat oxidation and mitochondrial function in rats fed a high-fat diet. *British Journal of Nutrition*.

[28] Birkenfeld A. L., Shulman G. I. (2014). Nonalcoholic fatty liver disease, hepatic insulin resistance, and type 2 diabetes. *Hepatology*.

[29] Greco M., Chiefari E., Montalcini T. (2014). Early effects of a hypocaloric, Mediterranean diet on laboratory parameters in obese individuals. *Mediators of Inflammation*.

[30] Adams L. A., Waters O. R., Knuiman M. W., Elliott R. R., Olynyk J. K. (2009). NAFLD as a risk factor for the development of diabetes and the metabolic syndrome: an eleven-year follow-up study. *The American Journal of Gastroenterology*.

[31] Hossain N., Afendy A., Stepanova M. (2009). Independent predictors of fibrosis in patients with nonalcoholic fatty liver disease. *Clinical Gastroenterology and Hepatology*.

[32] Fritsche J., Steinhart H. (1998). Analysis, occurrence, and physiological properties of trans fatty acids (TFA) with particular emphasis on conjugated linoleic acid isomers (CLA) - a review. *Lipid - Fett*.

[33] Whigham L. D., Watras A. C., Schoeller D. A. (2007). Efficacy of conjugated linoleic acid for reducing fat mass: a meta-analysis in humans. *The American Journal of Clinical Nutrition*.

[34] Blankson H., Stakkestad J. A., Fagertun H., Thom E., Wadstein J., Gudmundsen O. (2000). Conjugated linoleic acid reduces body fat mass in overweight and obese humans. *The Journal of Nutrition*.

[35] Steck S. E., Chalecki A. M., Miller P. (2007). Conjugated linoleic acid supplementation for twelve weeks increases lean body mass in obese humans. *The Journal of Nutrition*.

[36] Białek A., Tokarz A. (2013). Conjugated linoleic acid as a potential protective factor in prevention of breast cancer. *Postępy Higieny i Medycyny Doświadczalnej*.

[37] Smit L. A., Baylin A., Campos H. (2010). Conjugated linoleic acid in adipose tissue and risk of myocardial infarction. *The American Journal of Clinical Nutrition*.

[38] Ebrahimi-Mameghani M., Jamali H., Mahdavi R., Kakaei F., Abedi R., Kabir-Mamdooh B. (2016). Conjugated linoleic acid improves glycemic response, lipid profile, and oxidative stress in obese patients with non-alcoholic fatty liver disease: a randomized controlled clinical trial. *Croatian Medical Journal*.

[39] Shadman Z., Taleban F., Saadat N., Hedayati M. (2013). Effect of conjugated linoleic acid and vitamin E on glycemic control, body composition, and inflammatory markers in overweight type2 diabetics. *Journal of Diabetes & Metabolic Disorders*.

[40] Venkatramanan S., Joseph S. V., Chouinard P. Y., Jacques H., Farnworth E. R., Jones P. J. H. (2010). Milk enriched with conjugated linoleic acid fails to alter blood lipids or body composition in moderately overweight, borderline hyperlipidemic individuals. *Journal of the American College of Nutrition*.

[41] Onakpoya I. J., Posadzki P. P., Watson L. K., Davies L. A., Ernst E. (2012). The efficacy of long-term conjugated linoleic acid (CLA) supplementation on body composition in overweight and obese individuals: a systematic review and meta-analysis of randomized clinical trials. *European Journal of Nutrition*.

[42] Kraft J., Collomb M., Möckel P., Sieber R., Jahreis G. (2003). Differences in CLA isomer distribution of cow’s milk lipids. *Lipids*.

